# Supplementary material for: The interactome of intact mitochondria by cross-linking mass spectrometry provides evidence for coexisting respiratory supercomplexes
Source: Mol Cell Proteomics. 2017 Dec 8;17(2):216–32. doi: 10.1074/mcp.RA117.000470 (PMC5795388; doi:10.1074/mcp.RA117.000470)
Supplement: Supplemental Data [file supp_RA117.000470_133922_0_supp_23978_fzffwf.zip › spectra_annotation/mito_DR_spectra_annotation/448-1-6-1-11-1.pdf]

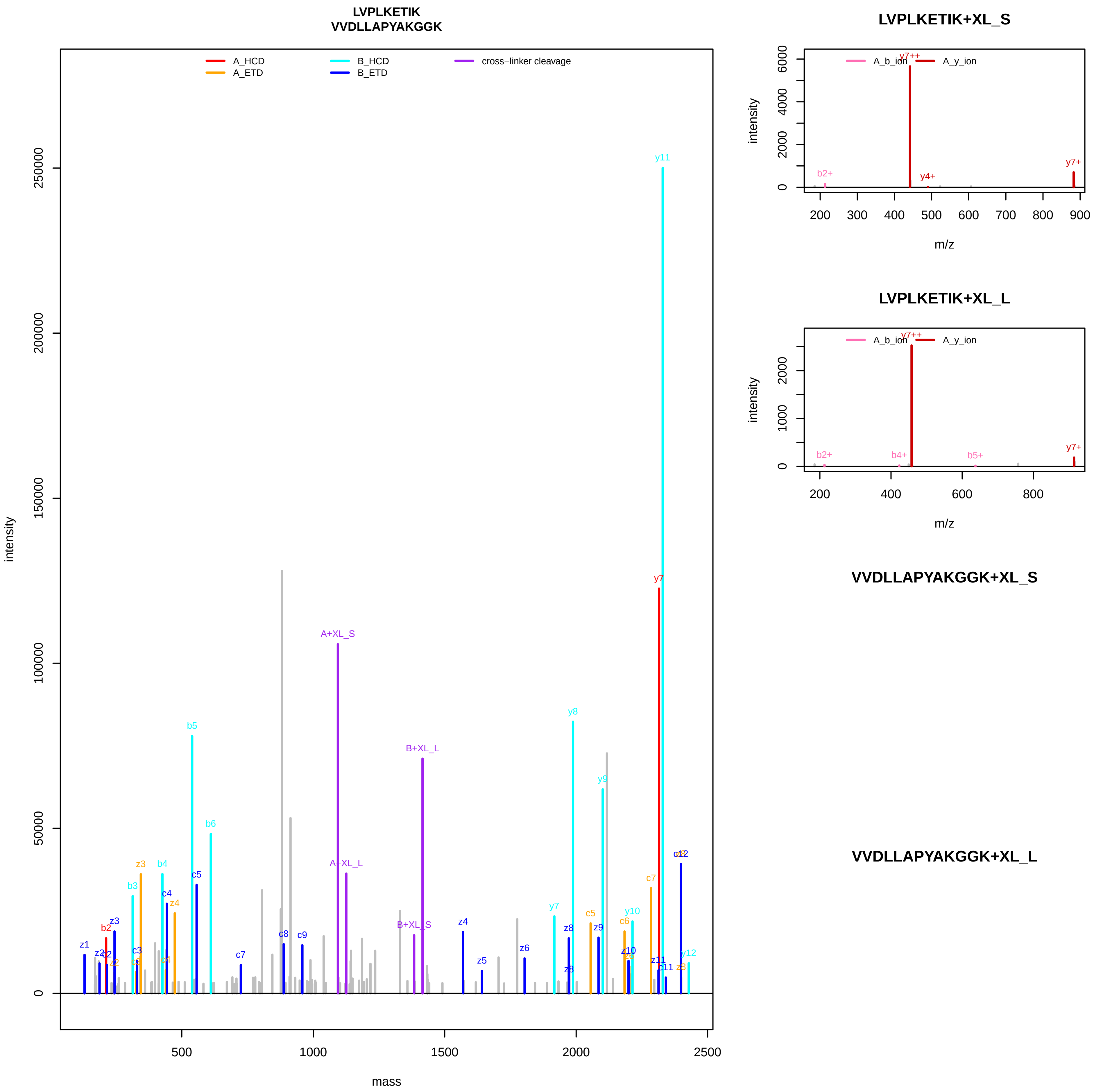
LVPLKETIK
VVDLLAPYAKGGK
A_HCD
A_ETD
B_HCD
B_ETD
cross−linker cleavage
intensity
mass
0
50000
100000
150000
200000
250000
500
1000
1500
2000
2500
y11
y7
A+XL_S
A+XL_L
B+XL_L
B+XL_S
y8
y9
b5
b6
b3
b4
z3
z4
c4
c5
b2
z1
c7
c8
c9
z4
z5
z6
y7
z8
z9
c5
c6
y10
z10
c7
c12
z11
c11
y12
LVPLKETIK+XL_S
A_b_ion
A_y_ion
y7++
b2+
y4+
y7+
m/z
0
2000
4000
6000
200
300
400
500
600
700
800
900
LVPLKETIK+XL_L
b4+
b5+
1000
VVDLLAPYAKGGK+XL_S
VVDLLAPYAKGGK+XL_L
